# Wastewater surveillance of open drains for mapping the trajectory and succession of SARS-CoV-2 lineages in 23 cities of Maharashtra state (India) during June 2022 to May 2023

**DOI:** 10.1016/j.heliyon.2025.e42534

**Published:** 2025-02-07

**Authors:** Sejal Matra, Harshada Ghode, Vinay Rajput, Rinka Pramanik, Vinita Malik, Deepak Rathore, Shailendra Kumar, Pradnya Kadam, Manisha Tupekar, Sanjay Kamble, Syed Dastager, Abhay Bajaj, Asifa Qureshi, Atya Kapley, Krishanpal Karmodiya, Mahesh Dharne

**Affiliations:** a National Collection of Industrial Microorganisms (NCIM), Biochemical Sciences Division, CSIR-National Chemical Laboratory (NCL), Pune, 411008, Maharashtra, India; b Academy of Scientific and Innovative Research (AcSIR), Ghaziabad, 201002, Uttar Pradesh, India; c Environmental Biotechnology and Genomics Division (EBGD), CSIR-National Environmental Engineering Research Institute (NEERI), Nehru Marg, Nagpur, 440020, India; d Department of Biology, Indian Institute of Science Education and Research (IISER), Pune, 411008, Maharashtra, India; e Chemical Engineering and Process Development (CEPD) Division, CSIR-NationaChemical Laboratory, Pune, 411008, Maharashtra, India; f Environmental Toxicology Group, FEST Division, CSIR-Indian Institute of Toxicology Research, 31 Mahatma Gandhi Marg, Lucknow, 226001, India

**Keywords:** Wastewater-based epidemiology, SARS-CoV-2, Open drains, WBE, Whole genome sequencing, COVID-19, LMIC

## Abstract

The timely detection of SARS-CoV-2 is crucial for controlling its spread, especially in areas vulnerable to outbreaks. However, due to a lack of sustainable and low cost methods, early detection of such outbreaks is impacting low to middle-income countries (LMICs). Leveraging Wastewater-Based Epidemiology (WBE), we examined the dissemination and evolution of the SARS CoV2 virus in open drains across urban, suburban and densely populated cities in selected regions in the state of Maharashtra, the third largest state of India. In the period from June 2022 to May 2023, 44.89 % of SARS-CoV-2 RNA were positive in RT-qPCR in wastewater samples collected from open drains across selected regions. Whole genome sequencing revealed 22 distinct SARS-CoV-2 lineages, with the Omicron variant, followed by the XBB variant, dominating, alongside other variants such as BF, BQ, CH, and BA.2.86, albeit with lower frequencies. Wastewater surveillance provided early insights into viral transmission, complementing clinical surveillance. Notably, our study detected emerging variants prior to clinical reporting, highlighting the potential of WBE for early detection. Findings underscore the correlation between population density and the trend of viral load. This study also highlighted the significance of using open drains for WBE as a low-cost, and sustainable tool, especially in LMICs, where adequate methods are lacking or difficult to deploy for accessibility.

## Highlights

• Early detection & monitoring of SARS-CoV-2 in open drains of Maharashtra, India.
• Correlation of population density with viral load trend.
• First report of wastewater-based BA -XBB transition from 2022-23, in Maharashtra.
• Early detection of VOCs such as BA.2.75, XBB1.16.X & BA.2.86.X in WW from open drain.
• Usage of open drains for WBE in LMICs.

## Graphical abstract

**Figure:**
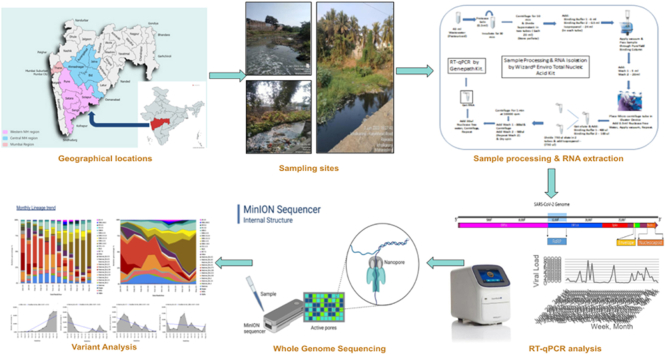

## Acronyms

SARS-CoV-2: Severe Acute Respiratory Syndrome coronavirus 2
WBE: Wastewater-Based Epidemiology
LMIC: Low- and Middle-Income Countries
WW: Wastewater
VOC: Variant of Concern
RT-qPCR: Real-Time Reverse Transcription Quantitative Polymerase Chain Reaction
Ct: Cycle Threshold
VTM: Viral Transport Medium
CDC: Centers for Disease Control and Prevention
IBSC: Institutional Biosafety Committee
RdRP: RNA-dependent RNA Polymerase
N: Nucleocapsid (gene)
E: Envelope (gene)
S: Spike (gene)
GISAID: Global Initiative on Sharing All Influenza Data
HIC: High-Income Countries

## 1 Introduction

The outbreak of the SARS-CoV-2 virus in December 2019, led to an acute respiratory illness called coronavirus disease 2019 (COVID-19) was initially reported in Wuhan, China, but subsequently evolved into a global pandemic [1,2]. The SARS-CoV-2 virus is an enveloped, single-stranded, having positive-sense RNA with a genome size ranging from 29.8 kb to 29.9 kb. It is classified under the genus *Betacoronavirus* and the subgenus *Sarbecovirus* [[3], [4], [5]]. COVID-19 spreads via direct contact with infected individual, fomites and through an inhalation of aerosolized droplets. Moreover, SARS-CoV-2 has been detected in the fecal samples of infected patients, indicating that oral-fecal transmission may also be a possible mode of transmission [4,6]. If the SARS-CoV-2 virus is inhaled, it enters the human host cell by binding its spike protein with ACE2 receptors present in pneumocytes in the bronchial epithelia [1]. Similarly, it enters the gastrointestinal cells by an ACE2 receptor-based mechanism. Its interferon-mediated immune response enriches opportunistic pathogens, resulting in inflammatory factors [7]. Consequently, individuals infected with SARS-CoV-2 virus often experience diarrhoea and shed viruses in sewage wastewater via feces [8]. Therefore, for our study, we selected open drains as the sampling sites for wastewater, as these are readily accessible in urban as well as in rural areas. Open drains carry wastewater from households, commercial establishments, hospitals and industrial facilities. Also, they map the wide geographical area, thus providing a representative sample of viral shedding for the larger population of a particular region. This can aid in identifying hotspots and prioritizing public health campaigns.

Wastewater-based epidemiology (WBE) was pioneered for typhoid fever surveillance in 1928 and poliomyelitis virus surveillance in the year 1939, roughly 60 years before the World Health Organization (WHO) precisely approved its formal endorsement for disease surveillance as a complementary tool to molecular and clinical diagnosis [[9], [10], [11]]. Following this, a variety of studies have been published highlighting WBE for pesticide exposure [12], population exposure to phthalate plasticizers [13] and monitoring of synthetic cathinone use [14], consumption of illicit stimulant drugs in Australia [15], Transmission lineages of Echovirus [16] etc. The reawakening of interest in WBE was triggered by the emergence of SARS-CoV-2 in year 2020, highlighting the critical intersection of environmental monitoring and clinical frameworks [17]. WBE can effectively signal the presence of SARS-CoV-2 variants within a community before they are clinically detected. So, it serves as a proactive measure to identify the spread of virus carried by humans asymptomatically [18]. Thus, WBE can set forth an early warning for the spread of disease in a particular region. Additionally, the surveillance of a large number of the population could become monotonous, laborious and expensive, which can be tackled by using WBE, making it easy to track infections during pandemics [19]. The COVID-19 pandemic's impact is challenging to evaluate due to limitations in traditional epidemiological indicators, like confirmed case counts and deaths, which are biased by limited testing and healthcare access, especially in low- and middle-income countries. Due to which the interpretation of the true spread of the SARS-CoV-2 virus can be underestimated. Also, the hospitalization data misses asymptomatic and mild cases [20]. Consequently, due to an incomplete understanding of community transmission, public health interventions cannot become absolutely effective, which are solely based on clinical testing data [21]. Therefore, this highlights the need for alternative data sources for a more accurate understanding of the spread of the virus in the community. So, WBE can be a more comprehensive and unbiased approach for monitoring viral trends, especially, in the areas with limited access to clinical testing. However, the studies detecting SARS-CoV-2 RNA in wastewater are mainly from high-income countries, with few from low- and middle-income countries (LMICs) like Brazil, India, Malaysia, Bangladesh and Indonesia [20,[22], [23], [24]].

In the present study, WBE was exploited for comprehensive surveillance of SARS-CoV-2 variants across 23 cities in the West Indian state of Maharashtra from June 2022 to May 2023. All the cities were strategically divided into three geographical regions (Mumbai, Central, and Western). The principal objective of this study was to evaluate the potential of WBE as a prescient indicator, capable of detecting emergent viral variants prior to their extensive dissemination within the community. Additionally, the study aimed to assess the ability of WBE to provide early warnings for public health responses, especially in the LMICs, by using open drains. Moreover, we hypothesized that wastewater surveillance could unveil potential discrepancies in the chronological onset of variant circulation throughout the state. This insight could contribute to tailored public health strategies based on regional needs.

## 2 Materials and methods

### 2.1 Wastewater sample collection

**Fig. 1:**
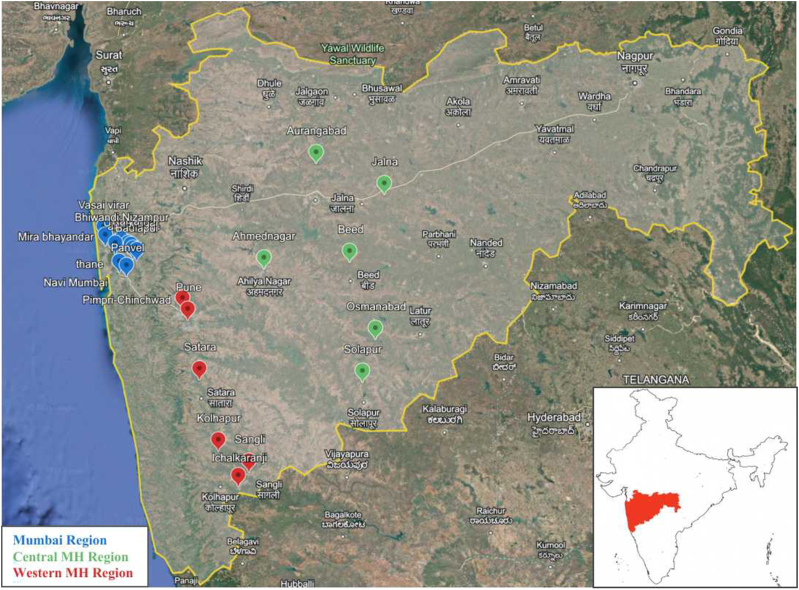
Geographical map representing the sampling location of all studied regions.

Wastewater sample collection adhered to established protocols outlined by the Centers for Disease Control and Prevention (CDC), USA (CDC, 2023) for COVID-19 wastewater surveillance. All the procedures were approved by the Institutional Biosafety Committee (IBSC), India. Samples were collected on a weekly basis from open drains in 23 cities throughout Maharashtra state (India), commencing in the third week of June 2022 and continuing through the fourth week of May 2023. To facilitate analysis and explore potential correlations between urbanization levels, population density, and variant emergence, the sampling sites were strategically divided into three distinct regions. The Mumbai region encompassed ten cities: Navi Mumbai (NM), Panvel (PNVL), Badlapur (BUD), Ambarnath (ABH), Ulhasnagar (ULNR), Kalyan-Dombivli (KYN), Bhiwandi Nizampur MC (BIRD), Thane (TNA), Mira Bhayandar (BY), and Vasai-Virar City MC (BSR). The Western Maharashtra region comprises six cities: Pune (PMC), Satara (STR), Sangli (SMK), Ichalkaranji (IKZ), Kolhapur (KOP), and Pimpri Chinchwad (PCMC). Finally, the Central Maharashtra region included Osmanabad (OBD), Jalna (JLN), Beed (BEED), Barshi (BRS), Solapur (SUR), Ahmednagar (AMN), and Aurangabad (AU) (Fig. 1). Around 500 ml of wastewater sample was collected from each location in sterile polypropylene bottles and transported to the laboratory in cold containers. **(**S Table 1**- Sampling sites details).** From 36 sampling sites, we collected a total of 1548 samples. From Mumbai region: 430, Central Maharashtra region: 559 and Western Maharashtra region: 559.

### 2.2 Wastewater processing and nucleic acid extraction

Sample bottles were wiped using 70 % ethanol and then were heat-inactivated at 60 °C for 1 h in a hot water bath (Julabo, USA). The Wizard® Enviro Total Nucleic Acid Kit (Promega Corporation, USA) was used for total nucleic acid extraction (TNA) as per the manufacturer's instructions. Briefly, the methodology was conducted in two steps. Initially, in the concentration step, 40 ml of wastewater sample was used to capture TNA directly on silica resin using a PureYield™ Midi Binding Column and eluted in 1 ml of nuclease-free water. In the purification step, 1 ml of concentrated TNA was purified using PureYield™ Minicolumn and finally, TNA was eluted in 60 μl of preheated nuclease-free water.

### 2.3 Real time with reverse transcription qPCR

Following extraction, real-time reverse transcriptase-quantitative polymerase chain reaction (RT-qPCR) was carried out for detection and quantification of SARS-CoV-2 nucleic acids using the DxCoViDx One v2.1.1 TK-Quantitative RT PCR kit (Genepath Diagnostics, India) as per the manufacturer's instructions. This kit is approved by the Indian Council of Medical Research (ICMR), India. The PCR reaction mixture was prepared by adding 9.25 μL of master mix, 0.75 μL of primer probe mix and 5 μL of RNA template in a total reaction volume of 15 μL. Reactions were run on the QuantStudio 5 Real-Time PCR System (Applied Biosystems/ThermoFisher, USA). Three independent SARS-CoV-2-specific targets, RNA-dependent RNA polymerase (RdRP) gene, Nucleocapsid (N) gene, and Envelop (E) gene were used for quantitative detection of RNA in the samples. According to kit instructions and guidelines, at least two out of three genes need to be present in the detectable range (Ct < 35) in the sample to be considered as positive for SARS-CoV-2. The COVID-19 Viral Load Calculation Tool (RUO) was used to quantify the SARS-CoV-2 viral load in the samples (https://coviquant.genepathdx.com/).

### 2.4 Library preparation and sequencing

A total of 1350 (From Mumbai region-400, Central Maharashtra region-489 and Western Maharashtra region- 461) SARS-CoV-2 RNA samples were reverse transcribed using “SuperScript™ IV VILO™ Master Mix with ezDnase” (ThermoFisher Scientific, USA). The resulting complementary DNA was used to amplify SARS-CoV-2 sequences with primer pools from the “Artic V5.3.2 NCOV-2019 panel” (Integrated DNA Technologies, USA). Then, amplicons were subjected to MinION nanopore sequencing using the SARS-CoV-2 virus PCR tiling – classic protocol (“SQK-LSK 109” with “EXP-NBD 196”) on the Mk1C device (Oxford Nanopore Technology, UK). Some of the samples were also sequenced on the Illumina NextSeq 550 sequencing platform, which were outsourced at the Next Generation Genomics facility at the Indian Institute of Science Education and Research (IISER), Pune, India. Sample libraries were prepared using Illumina Covid seq RUO kits (Illumina, USA). (https://sapac.illumina.com/content/dam/illumina-marketing/documents/products/datasheets/illumina-covidseq-test-data-sheet-1270-2020-008.pdf). Raw data of sequencing generated by Illumina and Nanopore was filtered and checked for quality using the Fastp program and the Nanoplot package respectively. BWA-Mem software was used to align high-quality reads with the reference SARS-CoV-2 genome (MN908947.3). Using SAMtools coverage/bedcov tools were used to obtain necessary alignment and coverage data for quality assessment. Only samples with 40 % genome coverage were considered for further downstream analysis. Using LCS software, the lineage composition of SARS-CoV-2 variants in each sample was estimated. LCS is a mixture model designed to determine the variant composition in environmental samples. LCS is designed to analyze complex sequencing data by leveraging lineage-specific mutations to identify the relative proportions of SARS-CoV-2 lineages. It begins with preprocessing, where raw reads undergo quality control and are mapped to a reference SARS-CoV-2 genome. Then, it identifies the mutations, including single nucleotide polymorphisms (SNPs) and Indels (insertions/deletions). Using a probabilistic decomposition algorithm, LCS assigns these mutations to their respective lineages, even in cases where mutations overlap between variants. This enables accurate quantification of the relative abundance of each lineage in the pooled sample [25].

## 3 Results and discussion

To effectively curb the spread of SARS-CoV-2 within communities, prompt detection of the virus is imperative because the traditional methods pose a challenge in detecting the emerging variants and accurately quantifying their prevalence [26]. Traditional methods, such as clinical testing and hospital-based surveillance, present several challenges to identify emerging variants and to accurately quantify viral prevalence. These tests majorly focus on symptomatic cases and underestimate the true infection rate due to asymptomatic cases [27]. Furthermore, delays in case reporting hinder the timely detection of outbreaks, reducing the effectiveness of public health interventions [26]. In light of this, our study utilized WBE to examine the virus's dissemination and evolution across urban, suburban and densely populated areas of Maharashtra state, areas that are particularly vulnerable to outbreaks. Drawing on the findings of Haque R. et al., which demonstrate the successful detection of SARS-CoV-2 in wastewater, unhindered by inhibitors, xenobiotics, diluted viral loads, and fragmented RNA [28]; we isolated SARS-CoV-2 RNA from wastewater samples. Subsequently, we employed RT-qPCR for viral load quantification and whole genome amplicon sequencing to understand the progression of SARS-CoV-2 lineages in the community.

Over a period of 12-month study conducted between June 2022 and May 2023, SARS-CoV-2 RNA was consistently detected in open drain wastewater samples collected from multiple cities across three major regions of Maharashtra: Mumbai (10 sites), Western Maharashtra (6 sites), and Central Maharashtra (7 sites). An RT-qPCR assay targeting three SARS-CoV-2 genes (N, RdRp, and E) was employed to screen all samples for SARS-CoV-2 detection and quantification. A total of 1548 samples were collected from WW open drain sites over the 12 months. Of these, 44.89 % (n = 695) were found to be positive for SARS-CoV-2 via RT-qPCR, whereas the remaining 55.11 % were negative. There could be several reasons for the high percentage of negative samples. The detection rates may be lowered by temporal variability, such as variations in COVID-19 prevalence and sampling during low transmission times [29]. Viral concentrations may also be reduced by dilution during wastewater transmission and rainfall [30]. Another reason can be the presence of inhibitory substances present in wastewater, such as organic matter, chemicals and detergents, which can interfere with the PCR amplification. This becomes a limitation to calculate the actual viral titre in the sample.

### 3.1 Quantification of SARS-CoV-2 RNA in wastewater

**Fig. 2:**
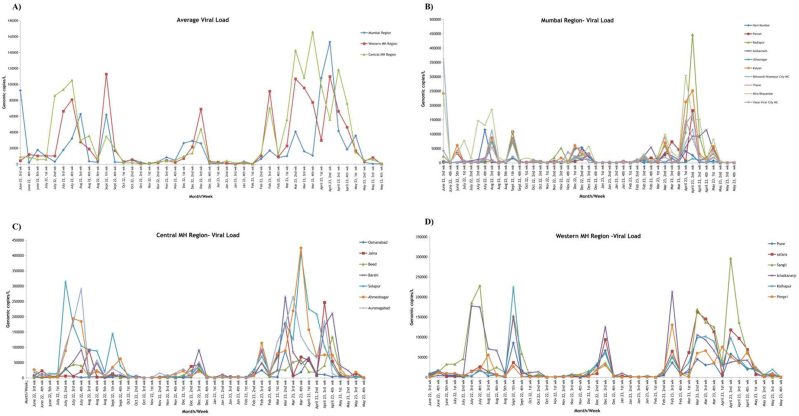
Weekly Viral load trend from June 2022–May 2023. - A) Average Viral Load, B) Mumbai region, C) Central Maharashtra region, D) Western Maharashtra region.

To further assess the extent of viral spread in the community across all regions, a viral load calculation tool (https://coviquant.genepathdx.com/) was employed to quantify SARS-CoV-2 RNA copies in each sample. Weekly analysis of viral loads across Mumbai, Western Maharashtra, and Central Maharashtra regions, including their respective cities, revealed almost identical viral load trend (Fig. 2A). Specifically, a significant surge in viral load was first detected in July 2022, which continued until September 2022. A second, less intense peak was remarked from November 2022 to December 2022. A third peak occurred from February 2023 to April 2023, declining from May 2023 onwards. Overall, the high detection rate of SARS-CoV-2 fragments across all the studied regions highlights the sensitivity of wastewater-based surveillance (WBS) for monitoring on-going viral activity within a population. This approach provides valuable insights into community-level transmission dynamics, potentially detecting trends that may not be fully reflected in clinical testing data. To understand the overall viral load trend of SARS-CoV-2 among all three regions, we considered factors such as population density and the efficacy of urban cities.

### 3.2 Mumbai region

Between July and August of 2022, the viral load increased, reaching its maximum of 187,000 gc/L. High viral titers was observed in Mira Bhayandar, Bhiwandi-Nizampur MC city. A second peak was observed from November to December 2022, with Kalyan recording the highest viral load (60,500 gc/L). A 3rd peak was initiated in February 2023 showing a gradual increase in viral load, which led to a peak in April 2023, with Badlapur reporting the highest titer in the region (447,000 gc/L) (Fig. 2B). Conclusively, a higher viral titer grabbed by Bhiwandi Nizampur MC, Mira Bhayandar, Kalyan and Ambarnath might be due to the high population density of the respective areas. These findings corroborate with previous studies which reported that the COVID-19 incidence rates are directly proportional to population density because viruses can be transmitted easily due to frequent human mobility and migrations within a particular area and the closer contact between population for a wider time span [24,[31], [32], [33]]. Hence, it can be inferred that the aforementioned locations were remarked with heightened viral loads in RT-qPCR analysis, likely attributed to their densely populated nature, which is prone to transmit the infections hastily. Similarly, the research by Panda et al. suggested that slums are conducive to the rapid spread of viruses due to their high density [34]. This study emulates our result in which cities such as Mira Bhayandar and Bhiwandi Nizampur Municipal Corporation (MC) city are characterized by substantial slum populations, exhibiting higher viral titers **(**S Table 3**- Population & Population Density details.).**

### 3.3 Central Maharashtra region

All the locations showed an increase in viral load from 1st week of July 2022. Among these highest viral titer was grabbed by Solapur (314,500 gc/L) followed by Aurangabad and Ahmednagar and gave rise to 1st peak. The 2nd peak in the region was exhibited in the 3rd week of Dec 2022 by Barshi with 206,000 gc/L. For the 3rd peak of the epidemic wave, viral load started increasing from the 1st week of Feb 2023, and the subsequent increase was noticed from the 1st week of Mar 2023. The peak was exhibited in the 4th week of March 2023 by Ahmednagar and Solapur with more than 400,000 gc/L of viral load (Fig. 2C). In this region, a weak correlation between population density and viral load was observed. Despite having higher population densities, no significant peaks of high viral load were noticed. This indicates that closer contact rates are influenced by both behavioural and environmental factors and the relationship between population density and contact rates is complex and varies across different geographical scales [35].

### 3.4 Western Maharashtra region

In the Western Maharashtra region, the first peak was recorded by Sangli (228500 gc/L). In Dec 2022, all 6 locations represented a 2nd peak with the highest viral titre of 68927 gc/L recorded by Ichalkaranji. In the 2 nd week of Feb 2023, a third epidemic wave was initiated with a gradual increase in genomic copies and grabbed a peak in the 3rd week of Feb 2023. For a couple of weeks, there was a drop in viral load and again in the 2 nd week of Mar 2023, the peak was noticed in all the locations, after which it subsequently reduced. Despite that, Sangli represented a steep increase to 296,500 gc/L in the 2 nd week of Apr 2023 (Fig. 2D). Overall, among all the cities of this region, despite having a smaller population but higher population density, Ichalkaranji and Sangli were noticed to exhibit higher viral titers during all three noticed peaks. It can be predicted that Sangli borders the metropolitan area of Pune (a city of the same region), which might be due to the facilities available in metropolitan cities with the easy accessibility of essential services like healthcare, food services, public transportation and retail. For that reason, there is a conceivability for observing the quick spread of pathogens causing higher viral loads in the locales due to the migration of people from suburban to urban areas [36].

[S Table 2- Weekly Viral Load (gc/L) of sampling sites (From 3rd week June 2022- 4th week May 2023)]

### 3.5 Resemblance in trends of SARS-CoV-2 viral load in wastewater and clinical cases

**Fig. 3:**
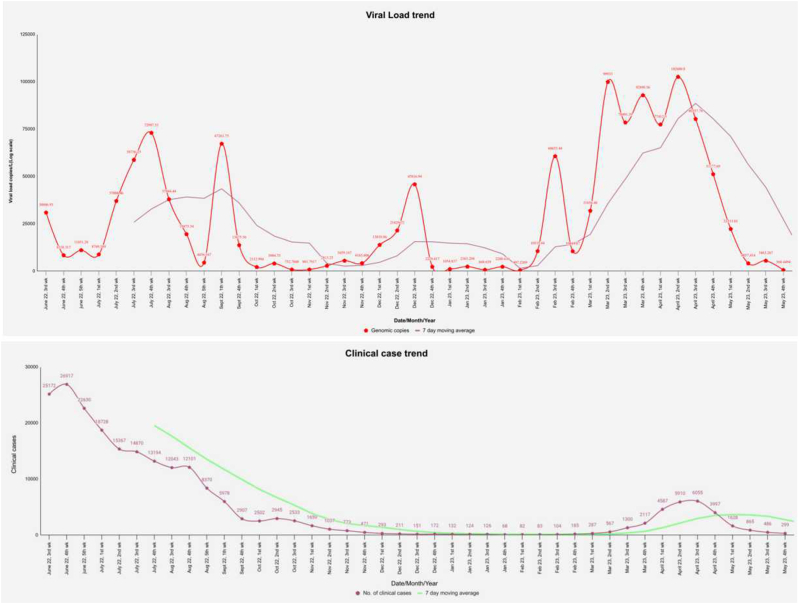
SARS-CoV-2 wastewater viral load trend compared to clinical cases in Maharashtra state during June 2022 to May 2023. A) Viral load trend in wastewater- Red line indicates genomic copies/L; Purple line indicates 7 day moving average. B) Clinical case trend in Maharashtra- Purple line indicates No. of clinical cases; Green line indicates its 7-day moving average.

The SARS-CoV-2 RNA viral load trend was compared with the positivity trend of the clinical cases in Maharashtra state. It was noticed from the clinical data obtained from IDSP (Integrated Disease Surveillance Program) that the number of clinical testing in the state drastically declined from the end of the year 2022 (Fig. 3). As of 2022, global COVID-19 reporting trends indicate that the true extent of infections and reinfections may be underestimated due to which numbers of clinical case reporting are declined [37]. Related observations were found in Clinical case Data from Maharashtra state from the year 2022; a significant decline in confirmed cases from nearly 26,000 cases in June 2022 and then down to about 5000–6000 cases in April 2023. Linking wastewater measurements to clinical cases is complex due to individuals' mobility across different neighbourhoods, which complicates tracing infections to specific areas based on residential addresses [38]. Even though, in the present study, during each peak of escalating viral load in wastewater, the number of clinical cases showed a slight height. In the initial period of study, the number of clinical case report was higher. However, the Omicron wave in August 2022 and ahead was distinct, primarily due to the variant's high transmissibility but low virulence and significant vaccination coverage among adults [28]. This combination resulted in less severe symptoms and reduced strain on critical care compared to earlier waves [27]. Consequently, it resulted in the less number of clinical tests in further years. Additionally, The SARS-CoV-2 virus load found in wastewater may be significantly influenced by seasonal changes. The stability of viral RNA in wastewater samples and viral transmission in populations can both be impacted by environmental variables such as temperature, humidity, and precipitation [39].

In our study, during the first peak of high viral load in June–July 2022, approximately 25,000 clinical cases were recorded. During the second peak, there were negligible clinical cases. However, during the transmission of the XBB variant, which marked the third peak of viral load, a slight increase in the number of clinical cases was observed, amounting to about 6000 cases [Supplementary Table 4**- Average genomic copies/L and number of clinical cases in Maharashtra (From year 2022**–**2023)].** Therefore, WBE is poised to become a key tool in tracking and managing the spread of COVID-19, offering a cost-effective and efficient alternative to individual testing. This approach can help pinpoint the spread and hotspots of the virus in communities, provided that the virus can be detected in feces [19]. However, there are some gains and losses of both clinical testing and WBE. The primary benefit of clinical testing is its capacity to offer precise, individual-level diagnosis, which enables focused therapy and the isolation of proven cases. However, there are some limitations, such as reliance on symptomatic individuals, unequal access to testing facilities; delays in reporting results etc. Conversely, WBE provides low cost non-intrusive ways to track transmission at community level. It can capture viral RNA from both symptomatic and asymptomatic individuals in the community. But it challenges such as dilution due to rainfall, difficulty in linking results to specific individuals and variability in viral shredding etc.

### 3.6 SARS-CoV-2 lineage speculation in wastewater

To acknowledge the variant trends over the state, we implemented extensive whole genome amplicon sequencing on the samples ascertained positive in RT-qPCR on both Nanopore (MK1C) and Illumina (Illumina NextSeq 550 sequencer) platforms. Among all the samples sequenced, 497 out of 989 samples passed on the Nanopore platform and 59 out of 361 samples passed on the Illumina platform, resulting in an overall sequencing success rate of 41.25 % with ≥40 % genome coverage. We successfully sequenced and analyzed a total of 556 samples which were passed in sequencing using the “Lineage decomposition” (LCS) tool to previse the lineage diversity and their relative probability. This comprehensive analysis identified 22 distinct lineages.

**Fig. 4:**
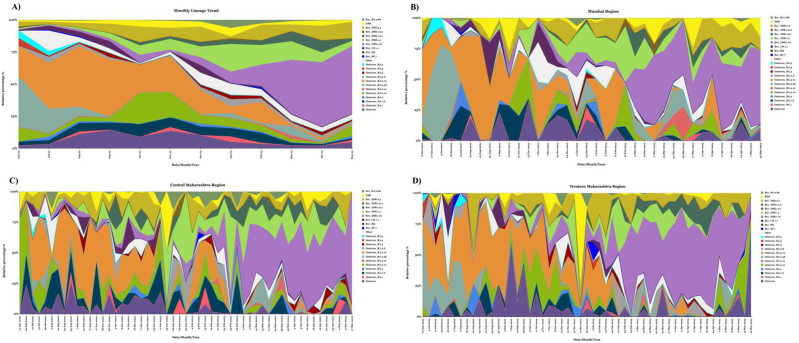
A) Monthly lineage trend of SARS-CoV-2 in wastewaters of Maharashtra (all studied regions) from June 2022 to May 2023; Lineage trends- B) Mumbai region, C) Central Maharashtra region, D) Western Maharashtra region.

Beginning from June 2022 to Dec 2022, the BA.2.75 lineage showed dominance, followed by BA.2.38 and BA.2.10. Further, XBB.1.16.X emerged as the most dominant from Dec 2022 to May 2023. Over this one year of study, XBB.1.16.X accounted for 24.7 %, followed by BA.2.75 with 16 % of total abundance. Besides this, other lineages such as BA.2.38 (5.3 %), BF.7.X (0.7 %), BQ.X (0.2 %), CH.1.1 (1.7 %), XBB (2.8 %), XBB.1.5.X (8.5 %) and BA.2.86.X (1.2 %), although less frequent, contributed to the overall diversity of SARS-CoV-2 variants observed in Maharashtra state. Our approach was to detect lineages outspreading in the regions of Maharashtra state that are more vulnerable to epidemic infections. In this study, when we investigated the reports of genome sequencing, three epidemic waves were observed: the 1st wave (3rd week of June 2022–2nd week of Sept 2022) and 2nd wave 1st week of Nov 2022–2nd week of Dec 2022) were noticed to be overlapping with the spread of BA.2.38 & BA.2.75. The 3rd wave (1st week of Feb 2023–1st week of May 2023) showed dominance of XBB 1.16.X. In this one year of surveillance, dominance was mainly shown by BA and XBB variants and their respective sublineages. Samal et al. also describe similar findings with the clinical reportings from Delhi, India [37,40]. In June 2022, the most dominant lineage was BA.2.38 with 39.6 % till August 2022 followed by BA.2.75 with 23.4 %. By July 2022, BA.2.75 had overtaken the dominance till Dec 2022. Thereafter, its eminence diminished and XBB.1.16.X demonstrated an upward trend, showing its supremacy by Jan 2023. In contrast, BA, BA.2.10, XBB.1.5.X and XBB.2.3.X were noticed to be signaled throughout a year of study (Fig. 4A). In all the three studied regions, the lineage pattern was perceived to be analogous with the dominance of BA.2.38, BA.2.75 and XBB.1.16. However, the lineage pattern of other variants varied, particularly in the Mumbai region.

### 3.7 Early detection of emerging variants

As we revealed the ascendancy of omicron sub-lineages such as BA, BA.2.38 and BA.2.75 during the initial months of study (June 2022–Dec 2022); in conformity with the GISAID database and some other published reports, BA.2.38 was clinically detected for the first time in India on Jan 07, 2022 [41]. While in Maharashtra state, it was clinically reported for the first time in Aug 2022. However, in the present study, BA.2.38 was noticed in wastewater in June 2022, which is two months prior to the clinical reporting. Similarly, BA.2.75 was globally reported for the first time in two states of India, namely in Karnataka and Jammu and Kashmir in June 2022, and when we started the present surveillance in June 2022, its prevalence was already there with 27.4 % of abundance and further increased to 43.8 % in Sept 2022. According to Karyakarte et al., a clinical case of XBB.1.16.X first appeared in India, in the sample collected from Tamilnadu state on Dec 25, 2023 [42]. On the other hand, as per the GISAID database and some Indian news collections, it was reported for the first time in India on Jan 9, 2023 [37]. While, in our report, some traces of XBB.1.16.X were observed in late July 22 that is 157 days earlier than clinical reporting with 9.9 % of total abundance in Ahmednagar city of the Central Maharashtra region, and subsequently increasing by Jan 2023, which finally reached 52.4 % in May 23 (Fig. 4C-lineage trend in central MH). Therefore, based on these findings, our study predicts that XBB may have been circulating in the community with low frequency prior to its high prevalence, which commenced in January 2023.

In the Mumbai region, a drastic increase in the abundance of XBB.1.16.X was observed by March 2023 (Fig. 4B). On the contrary, in the Central (Fig. 4C) and Western Maharashtra region (Fig. 4D), the same incidence was noticed from January 2023. Conclusively, 4/23 cities presented traces of XBB.1.16.X signals in Aug 2022 (Approx 5 months before). 6/23 cities revealed in Sept and Oct 2022 (4 months before). The rest of the cities showed its signals from Dec 2022 (1 month before). In addition to this, XBB.1.5.X was discovered in Africa on June 2, 2022, in Gujrat, India, on Oct 10, 2022; and in Maharashtra, India, it was reported clinically on Nov 23, 2022. While in this study its signals were noticed in the wastewater samples of the Mumbai region in June 2022, that is around 180 days before clinical prevalence (Fig. 4B). The BA.2.86 variant was first reported globally in the USA on Oct 22, 2022. Subsequently, it was identified in India on Oct 18, 2023, and a wastewater study in Thailand detected it earlier on July 28, 2023. Notably, our study detected signals of BA.2.86 as early as January 2023, where it constituted 4 % of the total variants identified with no documented clinical cases at that time. Similar findings were noticed for XBB.1.9.2, XBB.1.9.1.X and CH.1.1. Consequently, our study detects the transition of BA to the XBB variant from June 2022 to May 2023. Additionally, the dominating period of the variants detected in this report mirrors the GISAID database, expressing the early signals of the newly emerging variants in wastewater samples.

A similar study published earlier in Bangladesh [43] focused on WBE using sewer systems, which acknowledge the dilution and decay of viral RNA in wastewater. However, their study is limited to geographical context and sampling which is focused on only one isolation center in Noakhali. Whereas our study focuses on the wide geographical area for sampling. Also, the Previous studies by Rajput et al. and Dharmadhikari et al. reported Delta to Omicron transition and analysis of SARS-CoV-2 mutations using wastewater, respectively are restricted to only Pune city [23,41]. However, our study extends beyond the Pune region, encompassing the Western (including Pune city) and the Central part of Maharashtra state, including the Mumbai region. The three regions surveyed in this study encompass both urban and suburban areas. Notably, the urban cities within these regions, such as Navi Mumbai and Pune, exhibit a higher susceptibility to the transmission of new viral variants, attributable to their denser networks of transportation and the presence of multiple amenities for foreign visitors. This factor may contribute to a higher incidence of clinically reported cases in urban cities compared to suburban areas of these regions. Furthermore, the presence of new variants can be detected through wastewater surveillance in communities where clinical testing is limited. Rajput et al. suggested that the early detection and monitoring of infectious pathogens are crucial; thus, they advocate for the global implementation of WBE. They acknowledged that WBE is pivotal for tracking the dynamics of viral infections, as it allows for the early identification of viruses shed in wastewater, thereby serving as an essential tool for monitoring the spread of infections within communities [19,41].

WBE is a non-invasive method for monitoring SARS-CoV-2 in communities by analyzing sewage in near-real-time. However, the main challenge in applying WBE in resource-constrained settings that is in LMICs with the limited capacity to sample via a sewage network. Globally, in High Income Countries (HICs), centralized sewage systems are found, but, most areas, particularly from suburban regions in LMICs, lack centralized sewer connections, limiting the reach of WBS. This makes it an inapplicable approach for central sampling where large populations are unconnected to these systems and dispose of waste differently [44,45]. This necessitates sampling from water systems that involve environmental water sources like open drains, rivers, and surface water in which a limited number of populations, particularly from the smaller region among the community is involved for WBS. Interestingly, sampling from open drains could be called a targeted WBE that can be utilized to monitor viral spread in areas where outbreaks are more probable, such as institutions, hospitals, Pilgrimage destinations (in LMICs like India, Saudi Arabia, Jerusalem etc.) and other tourist gathering places. Therefore, to achieve an affordable, sustainable, and effective implementation of WBE, combining open drain sewage systems with centralized and decentralized approaches should be considered in LMICs that can help identify and assist hotspot areas [22,44]. Despite this, strategically placed sewage treatment sites can also lead to WBS and effectively monitor disease prevalence cost-effectively [38]. Moreover, the lack of formal sewerage systems in LMICs complicates wastewater surveillance, but adapting environmental surveillance methods for these areas could improve monitoring and response to COVID-19 and future pandemics [20]. This data can then be used for retrospective analysis to trace back the source communities [46], aiding in the early detection of COVID-19 infections up to several weeks before cases are clinically reported at a city-wide scale. The ethical ramifications of sampling drainages must be taken into account in addition to their practical benefits, which include cost-effectiveness and the capacity to collect a wider variety of viral loads from different sources [47]. Significant ethical questions are raised by wastewater sampling from sewers, especially concerning permission and privacy [48]. Wastewater analysis that targets certain groups or individuals may unintentionally reveal personal health information since the data may be connected to specific demographics or geographic areas without the subjects' knowledge or agreement [49]. This calls into question the propriety of carrying out this kind of surveillance as well as the possibility of misusing the data gathered [47]. Thus, in order to guarantee that wastewater-based epidemiology is carried out openly, responsibly, and with respect for individual privacy rights while still advancing public health initiatives, researchers and public health officials must establish strong ethical guidelines and frameworks [48,49] (Kuhlman & Barrett, 2021; D'Aoust et al., 2021).

## 4 Conclusion

The implementation of wastewater-based epidemiology (WBE) in Maharashtra state (India) has proven to be a critical tool in the early detection and monitoring of COVID-19 variants. By analyzing open drains from various urban and suburban locations, this study effectively identified the presence and spread of emerging variants like BA.2.38, BA.2.75, XBB1.16.X, CH.1.1, BF.7.X, BQ.X, and BA.2.86.X approximately six months prior to their detection through clinical means. These findings underscore the value of WBE not only as a supplemental surveillance tool but also as a primary indicator for potential outbreaks. In this context, the utility of WBE in LMICs highlights the usage of open drains to monitor the pathogen spread within the smaller as well as larger community and its potential to significantly enhance pandemic preparedness and response, offering a cost-effective, efficient, and capacious approach to public health monitoring that could be crucial in managing future infectious disease outbreaks.

## CRediT authorship contribution statement

**Sejal Matra:** Writing – original draft, Visualization, Validation, Methodology, Investigation, Formal analysis, Data curation, Conceptualization. **Harshada Ghode:** Writing – original draft, Visualization, Validation, Methodology, Investigation, Formal analysis, Data curation, Conceptualization. **Vinay Rajput:** Writing – review & editing, Visualization, Validation, Software, Formal analysis, Data curation. **Rinka Pramanik:** Writing – review & editing, Methodology, Investigation, Conceptualization. **Vinita Malik:** Methodology, Conceptualization. **Deepak Rathore:** Methodology. **Shailendra Kumar:** Methodology. **Pradnya Kadam:** Visualization, Methodology. **Manisha Tupekar:** Visualization, Methodology. **Sanjay Kamble:** Resources. **Syed Dastager:** Resources. **Abhay Bajaj:** Supervision, Conceptualization. **Asifa Qureshi:** Conceptualization. **Atya Kapley:** Supervision, Project administration, Funding acquisition, Conceptualization. **Krishanpal Karmodiya:** Validation, Data curation. **Mahesh Dharne:** Writing – review & editing, Validation, Supervision, Resources, Project administration, Funding acquisition, Conceptualization.

## Data availability

Sequencing data of wastewater samples have been submitted to NCBI under BioProject ID PRJNA1110039 and the BioSample database **(**S Table- 5**).**

Data for Population density: https://www.indiacensus.net/states/maharashtra/density (S Table- 3)

## Funding

This work was financially supported by 10.13039/501100001412CSIR, India (Grant No. MLP103926).

## Declaration of competing interest

The authors declare the following financial interests/personal relationships which may be considered as potential competing interests:Declarations of interest: none.
