# Supplementary material for: Wastewater surveillance of open drains for mapping the trajectory and succession of SARS-CoV-2 lineages in 23 cities of Maharashtra state (India) during June 2022 to May 2023
Source: Heliyon. 2025 Feb 7;11(4):e42534. doi: 10.1016/j.heliyon.2025.e42534 (PMC11876887; doi:10.1016/j.heliyon.2025.e42534)

**Detailed procedure to use Viral Load Calculation tool:**

The COVID-19 Viral Load Calculation Tool (RUO) (<https://coviquant.genepathdx.com/>)

- Access to the tool can be acquired from the GenepathDx Diagnostics (<https://www.genepathdx.com/>)

1. Input qPCR data: enter the data: Sample ID and Ct values of all the target genes (Ct values of RNA templates and controls). Data is to be uploaded in an excel file format.
2. After uploading a file, click on the “Quantitative control based calculations”.
3. Select RT-qPCR kit used. (In this study we used CoViDx One v2.1.1TK)
4. Specify the controls in a “Sample type” tab
5. Mention the quant limits.
6. Click on calculate Quant result to calculate Average PCR concentration, VTM concentration, Quant result and Final results (Positive, Negative or Inconclusive)
7. Export results in Excel file format.

**Example:**


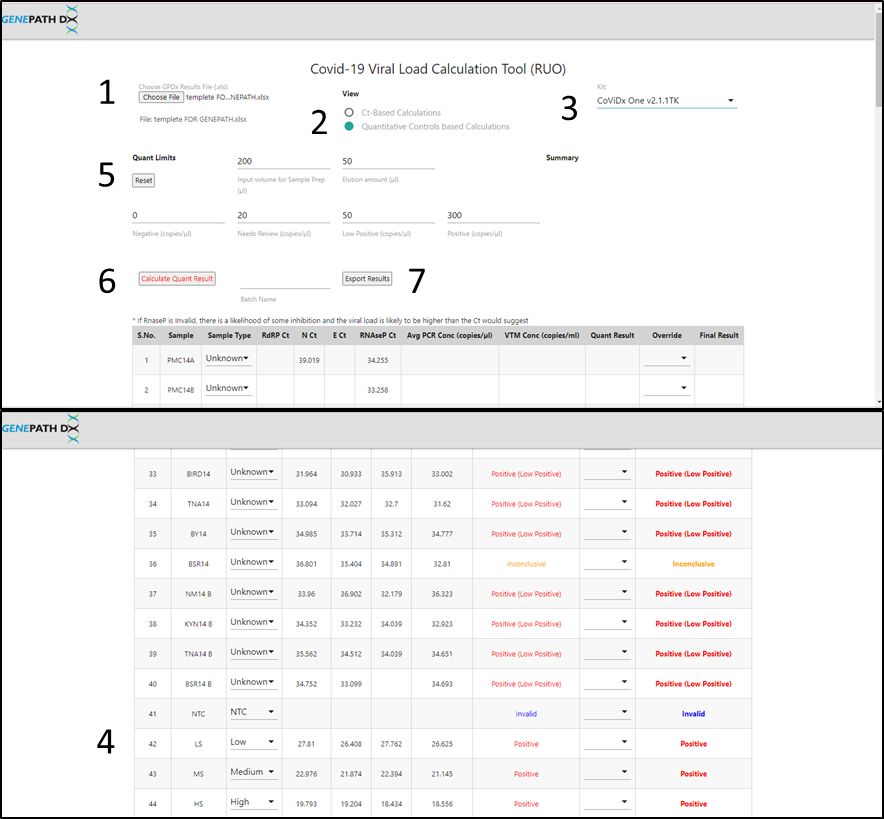


**Results Example:**


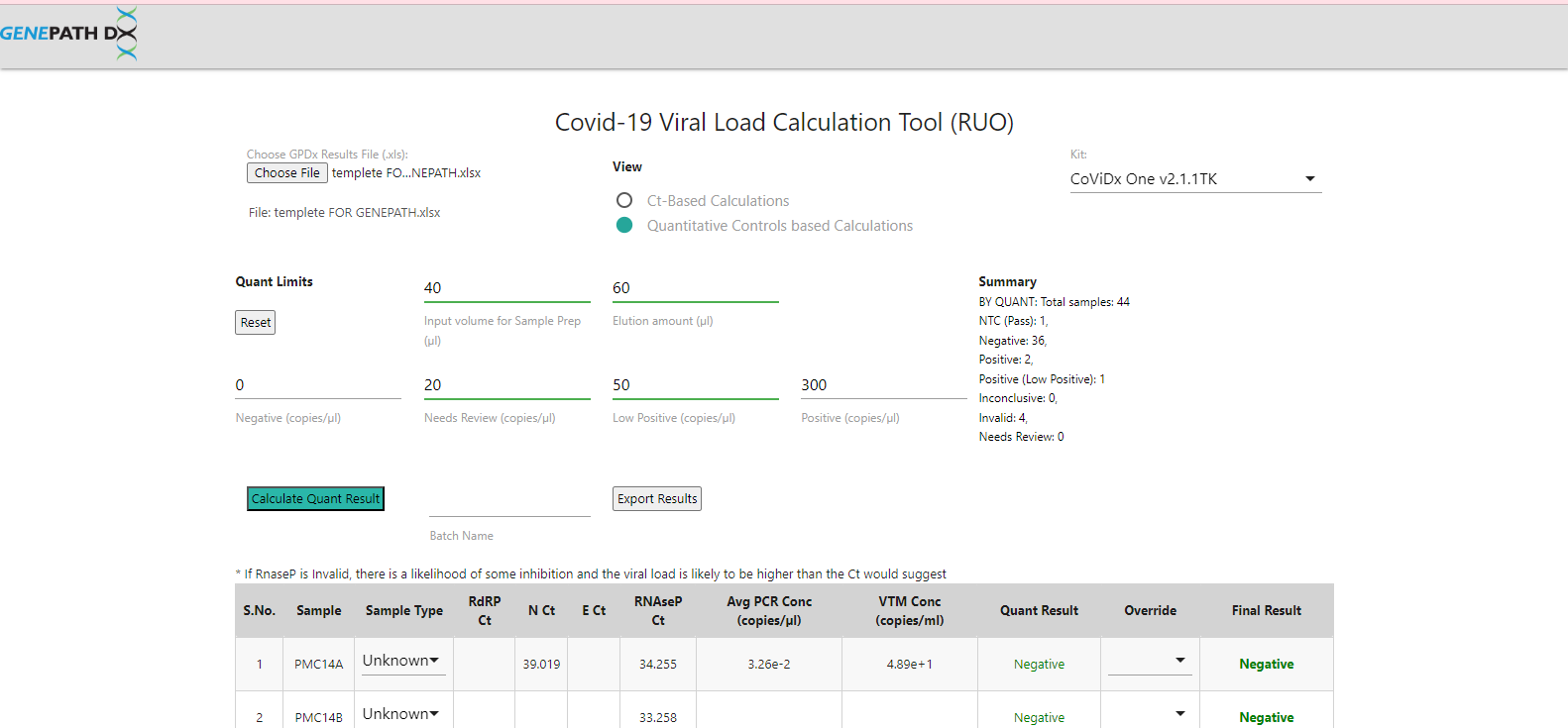


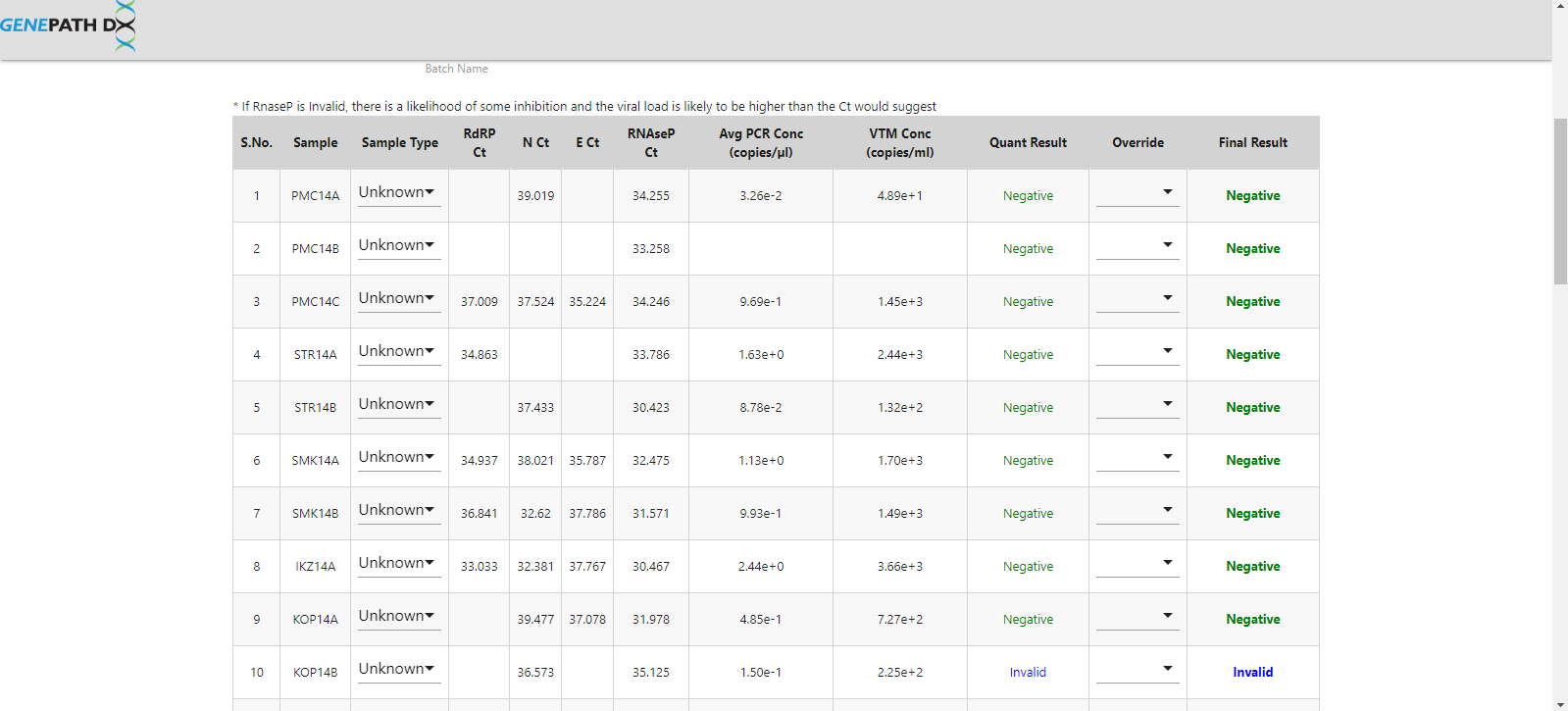


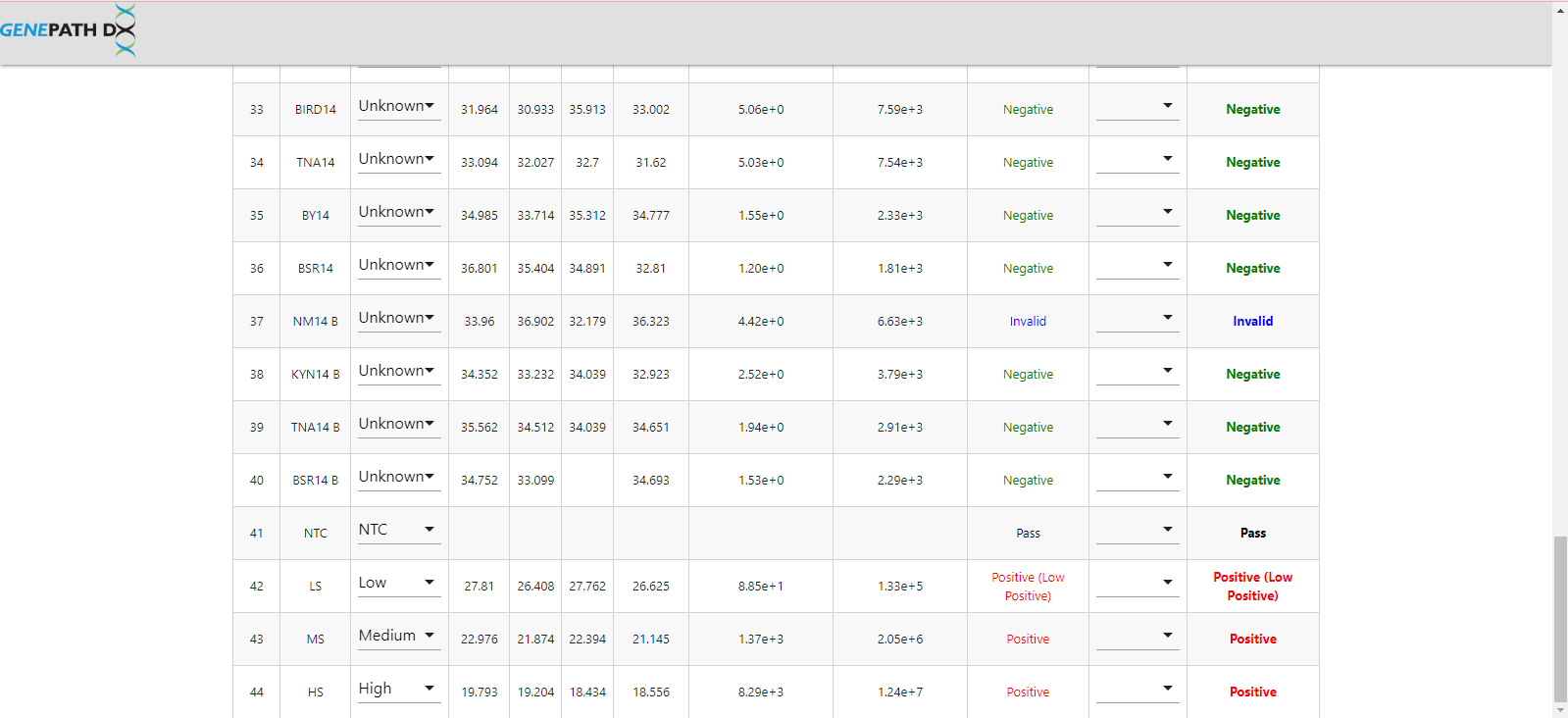

Supplement: Multimedia component 2 [file mmc2.docx]
